# Trimer stability of *Helicobacter pylori* HtrA is regulated by a natural mutation in the protease domain

**DOI:** 10.1007/s00430-023-00766-9

**Published:** 2023-05-14

**Authors:** Urszula Zarzecka, Nicole Tegtmeyer, Heinrich Sticht, Steffen Backert

**Affiliations:** 1 Division of Microbiology, Department of Biology, Friedrich-Alexander-Universität Erlangen-Nürnberg, Erlangen, Germany; 2 Department of General and Medical Biochemistry, Faculty of Biology, University of Gdańsk, Gdańsk, Poland; 3 Division of Bioinformatics, Institute of Biochemistry, Friedrich-Alexander-Universität Erlangen-Nürnberg, Erlangen, Germany

**Keywords:** HtrA, *Helicobacter pylori*, Trimer stability, Proteases

## Abstract

The human pathogen *Helicobacter pylori* is a major risk factor for gastric disease development. Serine protease HtrA is an important bacterial virulence factor that cleaves the cell junction proteins occludin, claudin-8 and E-cadherin, which causes gastric tissue damage. Using casein zymography, we discovered that HtrA trimer stability varies in clinical *H. pylori* strains. Subsequent sequence analyses revealed that HtrA trimer stability correlated with the presence of leucine or serine residue at position 171. The importance of these amino acids in determining trimer stability was confirmed by leucine-to-serine swapping experiments using isogenic *H. pylori* mutant strains as well as recombinant HtrA proteins. In addition, this sequence position displays a high sequence variability among various bacterial species, but generally exhibits a preference for hydrophilic amino acids. This natural L/S171 polymorphism in *H. pylori* may affect the protease activity of HtrA during infection, which could be of clinical importance and may determine gastric disease development.

**Supplementary Information:**

The online version contains supplementary material available at 10.1007/s00430-023-00766-9.

## Introduction

The bacterium *Helicobacter pylori* colonizes the stomachs of about half of the global population. If not treated, e.g. by antibiotic therapy, *H. pylori* can persist in most infected patients throughout their lives. While the majority of colonized individuals remain asymptomatic, infections can trigger the development of severe pathologies, including peptic ulcer disease, MALT lymphoma and stomach adenocarcinoma, in a subset of patients [1–3]. Several *H. pylori* virulence genes have been reported to impact gastric disease outcome [4–6]. Virtually all strains carry the *vacA* toxin, but isolates possessing the *vacA* s1m1 allele exhibit higher cytotoxicity [7]. The *cag* pathogenicity island (*cag*PAI) encoding a type IV secretion system (T4SS) and the effector protein CagA are only present in highly virulent strains and increase the risk of developing severe gastritis and malignant changes [1, 8]. The *cag* T4SS forms a membrane-anchored protein delivery device for CagA into the gastric epithelial cells [9]. Disruption of cell-to-cell junctions was commonly observed during *H. pylori* infection and bacterial entry into the gastric epithelium is facilitated by the *cag*PAI-independent serine protease HtrA (High-temperature requirement A) [10]. *H. pylori* HtrA is composed of an N-terminal signal peptide, followed by the catalytic protease domain and two C-terminal PDZ domains (PDZ-1 and PDZ-2) that serve as protein–protein interaction modules and provide substrate specificity [11–15]. HtrA is a periplasmic protein with both proteolytic and chaperone functions in the bacteria, but it is also secreted into the culture supernatant [11–13]. *H. pylori* HtrA assembles into various oligomers with a trimer as the basic functional building unit [12, 14]. Sedimentation velocity ultracentrifugation studies of HtrA oligomer assembly and stability displayed HtrA from *H. pylori* strain 26695 as a mixture of trimers, hexamers, and nonamers, whereas HtrA from strain N6 mainly formed trimers and 18-mers [12]. Moreover, structural analyses indicated that HtrA lacking the entire N-terminal part produces monomers, but no trimers, suggesting that the N-terminal region next to the signal peptide is essential in the formation of HtrA homotrimers [14]. Interestingly, in addition to trimer formation, N-terminal deletion of HtrA completely abolished the proteolytic activity, including cleavage of the model substrate β-casein [14]. Indeed, casein zymography suggested that the HtrA monomers missing the N-terminus are proteolytically inactive [15]. In contrast, mutation of the PDZ regions in HtrA had a different effect: deletion of the PDZ-2 region abolished cleavage of the cell junction protein E-cadherin, but did not affect processing of β-casein [14].

A quantification study revealed that *H. pylori* actively secreted about 9600 HtrA molecules per bacterial cell in a time course of eight hours, suggesting that HtrA represents a major virulence factor of the pathogen [16]. Importantly, secretion of HtrA by the bacteria during infection in vivo or experiments with the recombinant proteins in vitro resulted in cleavage of the epithelial cell junction components E-cadherin, occludin, and claudin-8, which opens the tight and adherens junctions, respectively [9, 17–19]. This allows paracellular transmigration of the bacteria between neighbouring gastric epithelial cells, and subsequent injection of CagA at basolateral compartments [9]. The *htrA* gene belongs to the core genome and is present in all *H. pylori* strains analysed to date [13, 20, 21]. However, it is unknown whether HtrA exhibits genetic polymorphisms and can differentially contribute to gastric disease development. Here, we report a natural mutation in the *htrA* gene resulting in a crucial amino acid change at position 171 that is associated with protein trimer stability. This may indicate an important role of this mutation in the process of inducing permanent colonization of *H. pylori* in humans.

## Materials and methods

### Plasmids and bacterial strains

Bacteria and plasmids used in this study are listed in Table S1 [22–36].

### Plasmid constructions

Plasmids pHJS5 and pUZN10 contained the *htrA* wild-type (wt) gene from *H. pylori* strains 26695 and N6, respectively, fused with His6-tag at the C-terminus and *E. coli* pelB signal sequence at the N-terminus. These plasmids were used as templates for constructing plasmids pUZ20 and pUZN22, in which we exchanged the leucine at position 171 for serine, or serine at position 171 to leucine by site-directed mutagenesis using PrimeSTAR GXL DNA Polymerase (Clontech, Takara). The nucleotide substitutions were verified by Sanger sequencing (Eurofins Genomics, Ebersberg, Germany). The primers for the construction of plasmids pUZ20 (primers P1, P2), and pUZN22 (primers P3, P4) are listed in Table S2.

### In vivo *H. pylori* mutagenesis

*HtrA* from *H. pylori* was mutated by gene replacement via double crossing-over recombination. This method was described earlier [13, 37] and resulted in the generation of functional *htrA* genes. The Δ*htrA* deletion mutation containing an *aphA-3* cassette (conferring kanamycin resistance) was replaced by mutated *htrA* from plasmids pUZ20 or pUZN22 along with a chloramphenicol resistance cassette using gene splicing by overlap extension PCR (SOE-PCR). First, we prepared three gel-purified DNA fragments amplified by PCR using primers and templates shown in Fig. S1. Next, a PCR with the two terminal primers P9 and P10 and three DNA fragments produced the spliced PCR (S-PCR) fragment. Finally, the purified S-PCR fragment was used (approx. 700 ng) to transform *H. pylori* N6∆*htrA* strain by natural transformation, followed by the selection of strains containing the corresponding *htrA* mutation at the correct chromosomal location. The introduced nucleotide substitutions were verified by Sanger sequencing (Eurofins Genomics, Ebersberg, Germany). Primers, templates, and PCR fragments are shown in Fig. S1 and summarized in Supplementary Tables S2 and S3. All wt and *htrA* mutated *H. pylori* strains used in this work are depicted in Fig. S2.

### *H. pylori* growth conditions

After revival from frozen stocks, the *H. pylori* wt strains and the N6 derivatives were cultured for two days at 37 °C under microaerobic conditions produced by sachets (CampyGen) in 2.5 L anaerobic jars (Oxoid, Wesel, Germany) on the GC agar (Oxoid, Wesel, Germany) supplemented with 10% donor horse serum (Biowest, Nuaillé, France), protease peptone (Oxoid, Wesel, Germany), vitamin mix (1%), and antibiotics: vancomycin (10 µg/mL), trimethoprim (5 µg/mL), amphotericin (4 µg/mL) and colistin (10 µg/mL), as described [12, 26]. To allow for selection, the media were supplemented additionally with chloramphenicol (8 µg/mL). The cells were suspended in the BHI medium (Oxoid, Germany), and the number of bacteria was evaluated by measurement of the optical density at 600 nm (OD_600_). To investigate the survival of *H. pylori* the number of colony-forming units (CFU) was determined by plating serial dilutions of bacterial suspensions as described [12]. The various temperatures (39 °C and 41 °C) and pH (pH5.2) stress conditions were generated as described [12, 31]. All experiments were performed in triplicate.

### Bacterial growth curve analysis

*H. pylori* culturing was basically carried out under conditions that were previously described in the literature [38]. *H. pylori* grown on plates were resuspended in BHI liquid broth medium with 10% FCS to an OD_600_ of 0.1, and then sub-cultured in 96-well microtiter plates (clear, flat-bottom, NEST Biotechnology Co.). Plates were incubated at 37 °C, 10% CO_2_, 5% O_2_, 500 rpm in a plate reader (Clariostar, BMG Labtech) with an atmospheric control unit (BMG Labtech). OD_600_ was automatically measured every 30 min until the stationary phase was reached. Growth curves were analyzed and processed using the MARS Data Analysis software 4.20 (BMG Labtech).

### Expression and purification of the HtrA proteins

The *E. coli* strain BL21(DE3) transformed with pHJS5, pUZN10, pUZ20, or pUZN22 (plasmids containing *htrA* genes) were used to overproduce recombinant HtrA variants with the C-terminal His tag (6x) in the pET vector System (Novagen, San Diego, CA, USA). Bacteria were grown at 37 °C in Luria–Bertani (LB) broth supplemented with kanamycin (50 µg/mL) to OD_600_ of 0.7; then the expression of *htrA* was induced by addition of 0.5 mM isopropyl-β-D-thiogalactopyranoside (IPTG) for 2–3 h (37 °C). After lysis and clearing of the lysates by centrifugation, all HtrA proteins were purified through nickel-affinity chromatography under native conditions as described previously [12].

### SDS-PAGE, immunoblotting, and casein zymography

The SDS buffer was mixed with bacterial pellet or recombinant proteins and boiled for 10 min. Samples were separated by SDS-PAGE. The separated proteins were stained by Coomassie Brilliant Blue (Merck, Darmstadt, Germany) or blotted onto polyvinylidene difluoride (PVDF) membrane (Carl Roth, Karlsruhe, Germany), followed by blocking and incubating the blotting membrane with the appropriate antibodies in 5% non-fat dry milk in TBS-T buffer (200 mM Tris pH 7.4, 1.4 M sodium chloride and 1% Tween-20) [39]. For protein detection, the following primary antibodies were incubated with membranes for 1.5 h at room temperature: mouse monoclonal α-His-Tag (Proteintech, Manchester, UK, cat. #66005), rabbit polyclonal α-HtrA [13], rabbit α-UreB antibody (using the conserved peptide HDYTIYGEELK as antigen). The horseradish peroxidase-conjugated secondary antibodies goat α-rabbit and goat α-mouse IgGs (Thermo Fisher Scientific, cat. #31462 and cat. #31446) were used in combination with the ECL ™ Prime Western Blotting kit (GE Healthcare Chicago, IL, USA) to detect bound antibodies. Casein zymography was performed as described [12, 15] and shown in Fig S3. The appropriate amounts of recombinant protein or bacterial lysates of *H. pylori* were added to SDS- casein buffer without β-mercaptoethanol (final concentration 46 mM Tris pH 6.8, 7.4% glycerol, 2.2% SDS, 0.007% bromophenol blue) and were incubated 20 min at room temperature. The samples were separated into 10% SDS-PAGE gels containing 0.1% casein (Carl Roth, Germany). For protein renaturation after gel electrophoresis, the gels were incubated in renaturation buffer (2.5% Triton-X100) for 60 min at room temperature, with the buffer changed every 30 min, to remove the SDS from the gel and from the protein-SDS complexes. Given that HtrA has chaperone functions, the removal of SDS from the proteins initiated the recovery of the native HtrA structure and restored HtrA activity, as shown before [11, 12, 15, 17, 31, 43, 44]. After protein renaturation, casein cleavage was performed for 16 h at 37 °C. To investigate the effect of SDS concentration on HtrA trimer stability, SDS-casein buffer with different SDS concentrations (5; 2.2; 1; 0.1%) was prepared and conventional SDS-PAGE running buffer was used.

### Analysis of HtrA proteolytic activity

The analysis of proteolytic activity of *H. pylori* HtrA against β-casein has been described [12], and the cleavage profile was recently determined [40]. In brief, 0.24 μM recombinant HtrA was mixed with 10 μM β-casein in 50 mM HEPES pH 6.2, 100 mM NaCl and incubated at 37 °C for 90 min in a final volume of 200 μL. Samples without HtrA were used as controls. The reaction was terminated by the addition of Laemmli lysis buffer (30 mM Tris-HCl, pH 6.8, 5% glycerol, 1.5% sodium dodecyl sulfate (SDS), 0.005% bromophenol blue) and freezing at − 20 °C immediately after boiling. The samples were then resolved in 15% SDS-PAGE and the gels were stained with Coomassie Brilliant Blue.

### Analysis of HtrA sequence variations from various bacterial species

HtrA proteins from different bacterial species were examined using sequences obtained from the UniProt database and available full genome sequences. The hydrophobicity of amino acid residues was analysed using the Praline tool of the IBIVU server [41, 42].

### Statistics

All data were generated from at least three independent experiments. The error bars in the corresponding figures represent the standard deviation values or the standard error of the mean (SEM). Significant differences were analysed using the Bonferroni test (ns- no significant differences; ***p < 0.001; **p < 0.01). The areas of the casein gels corresponding to the position of the HtrA trimer was examined densitometrically using a 1DScan EX program (Scanalytics Inc., United States).

## Results

### Clinical *H. pylori* strains exhibit HtrA in two fractions as analysed by casein zymography

**Fig. 1:**
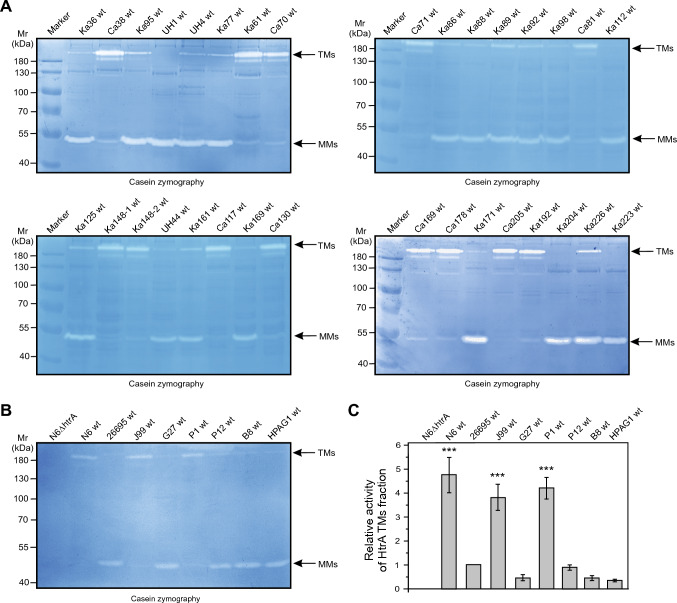
Mutation at amino acid position 171 causing changes in HtrA trimer (TM) stability in 40 clinical *H. pylori* strains. **A** Casein zymography showing HtrA proteolytic activity and TM stability from 32 indicated clinical *H. pylori* wt strains. MMs denotes the band of the monomers. **B** Proteolytic activity and TM stability from 8 fully sequenced *H. pylori* wt strains with known *htrA* genes. **C** Densitometrical quantification of the relative activity of HtrA TMs in panel (**B**)

We used casein zymography (described in Fig. S3) to study the production of proteolytically active HtrA proteins by 40 *H. pylori* wild-type (wt) isolates from our strain collection (Table S1). Two main forms of caseinolytically active HtrA were displayed in polyacrylamide gels as white bands, indicating complete digestion of the casein substrate. A proteolytic band at the size of ~ 55 kDa corresponded to the monomeric form (hereafter MMs) of the protein; the oligomeric fraction at ~ 180 kDa presumably consisted of trimers (hereafter TMs) (Fig. 1A, B). The latter fraction comprised stable TMs that did not dissociate into MMs during electrophoresis in the presence of SDS. In contrast, monomeric HtrA likely originated from conformationally less stable TMs that dissolved into MMs during electrophoresis in the presence of SDS (SDS-PAGE). Interestingly, although N-terminally truncated MMs of HtrA were previously proposed to be proteolytically inactive [15], caseinolytic activity was observed for full-length HtrA in both the TM and MM fractions. Thus, in contrast to N-terminally truncated HtrA, full-length HtrA MMs might either possess residual caseinolytic activity or the MM caseinolytic bands were generated by HtrA molecules that reassembled into TMs after a renaturation step. Our experiments included eight well-known *H. pylori* strains, whose genomes are fully sequenced with available *htrA* genes in the Genbank database, such as N6, 26695, J99, G27, P1, P12, B8 and HPAG1 (Fig. 1B). Interestingly, while HtrA of strains N6, J99 and P1 predominantly formed stable TMs, HtrA of strains 26695, G27, P12, B8 and HPAG1 was mainly presented in the MM fraction (arrows). The zymograms and the corresponding quantification of the active HtrAs in the TM fraction of each strain (Fig. 1C) suggest that *H. pylori* isolates produce HtrA either predominantly as MMs or as TMs, depending on the strain tested. As expected, an *htrA* knockout mutant [13], used as control, did not show proteolytic activity (Fig. 1B, lane 1).

### TM stability of *H. pylori* HtrA correlates with a natural mutation in the protease domain

**Fig. 2:**
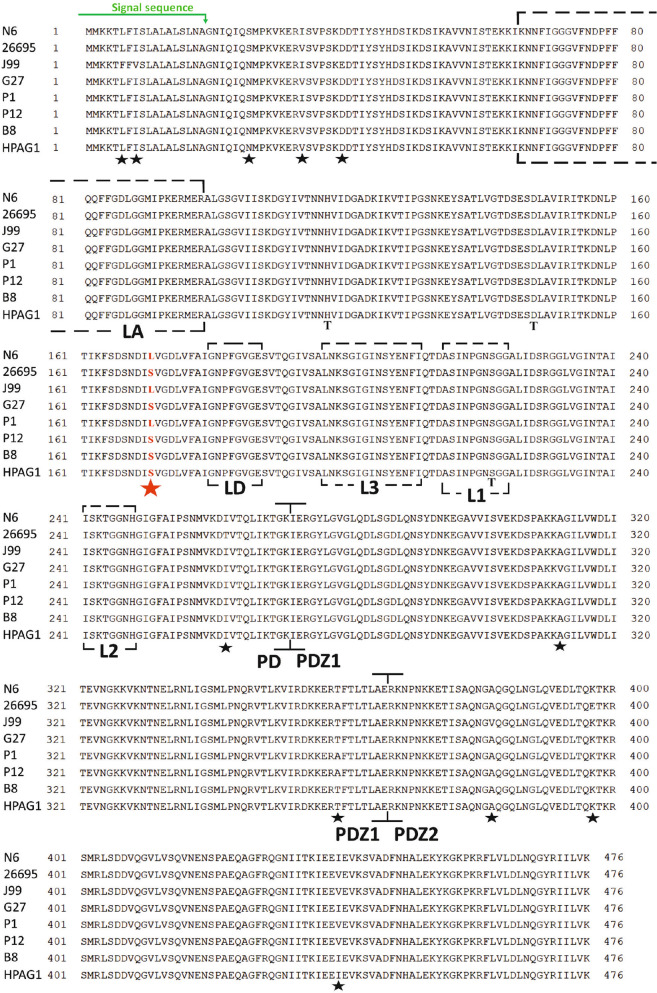
Alignment of the HtrA amino acid sequences from selected *H. pylori* strains—N6, 26695, J99, G27, P1, P12, B8 and HPAG1. The important regulatory loops (LA, LD, L1, L2, L3) as well as the domain organisation of HtrA are marked with dashed lines. PD—protease domain, T-catalytic triad. Black asterisks indicate the position of variable amino acid residues among the sequences. Amino acid position 171 in the protease domain of HtrA was labelled with a red asterisk

To investigate whether the generation of HtrA bands as MMs or TMs is genetically determined, we compared the *htrA* genes and resulting proteins from strains N6, 26695, J99, G27, P1, P12, B8 and HPAG1. Alignment of these HtrA sequences revealed 12 mutations across the entire gene that resulted in amino acid changes (Fig. 2, marked with asterisks). One of these mutations that was localized in the protease domain of HtrA, resulted in an amino acid change at position 171, encoding either a leucine (L) or a serine (S) (Fig. 2, marked with a red asterisk). Analysis of the hydrophobicity of the amino acids showed a significant difference between leucine (hydrophobic) and serine (hydrophilic) residues, which may play a role in establishing specific protein structure features, interactions, and functions (Fig. S4). Furthermore, this mutation was the only one in the eight sequences correlating with TM stability, while the other mutations exhibited a non-consistent distribution. The abundance of stable TMs in strains N6, J99 and P1 correlated with HtrA possessing a 171L allele, whereas mostly MMs were detected for strains 26695, G27, P12, B8, and HPAG1 that all contained the 171S mutation (Figs. 1 and 2). These findings suggest that position 171 may be important in facilitating TM stability of HtrA.

### TM stability of HtrA is determined by amino acid position 171 in isogenic *H. pylori* mutants and recombinant proteins

**Fig. 3:**
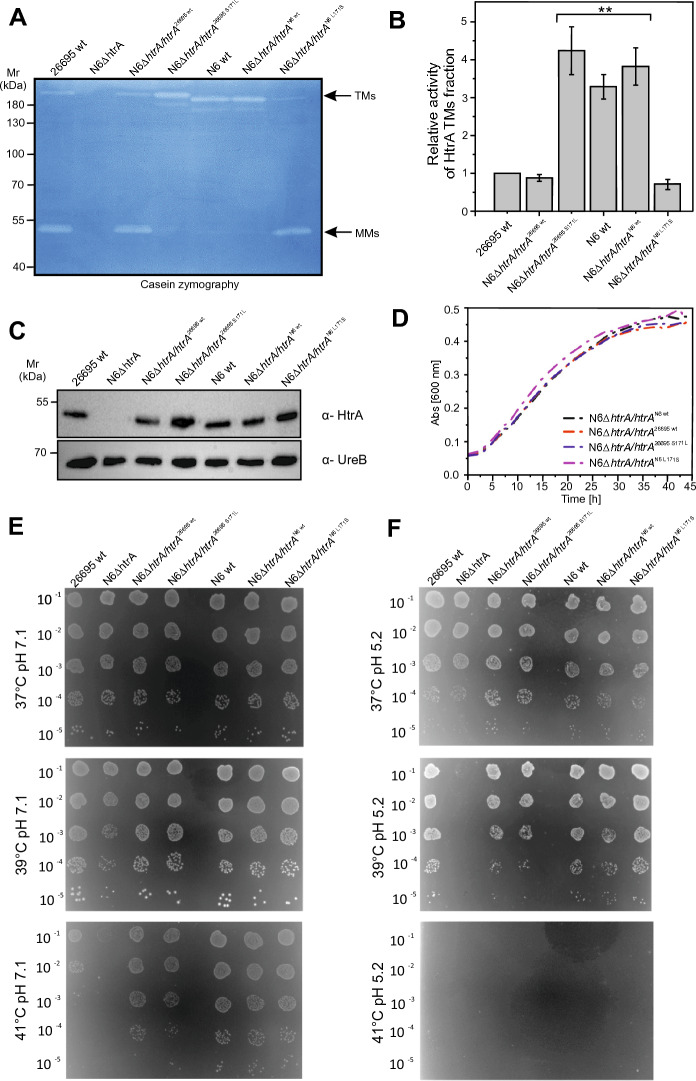
Mutation analysis in *H. pylori* bacteria causing changes of HtrA TM stability. **A** HtrA proteolytic activity and **B** quantification of the relative amounts of HtrA with substituted amino acids (L or S) at position 171. The error bars represent the standard error of the mean (SEM) from five independent experiments. Significant differences compared to 26695 wt were analysed using the Bonferroni test. **C** Western Blot analysis of HtrA and UreB levels in the tested *H. pylori* strains, which were grown on GC agar plates supplemented with 10% horse serum. **D** *H. pylori* strains were grown in liquid medium (BHI/10% FCS) in a microtiter plate at 37 °C, 5% O_2_, 10% CO_2_. **E**, **F** Growth properties of *H. pylori* bacteria, which were cultivated for 5 days under the indicated stress conditions

To investigate the impact of 171L or 171S on TM formation and stabilization of HtrA, we produced a set of isogenic HtrA mutant strains by PCR-based mutagenesis (Figs. S1 and S2). In particular, the 171L HtrA of strain N6 was mutated to 171S (*htrA* N6^L171S^), and the 171S HtrA wt allele of strain 26695 was changed to 171L (*htrA* 26695^S171L^). The corresponding casein zymograms revealed that all 171L-type HtrAs efficiently formed stable TMs. In contrast, HtrAs containing 171S were predominantly present in the MM fraction indicating that the assembled TMs were less stable than those possessing 171L and dissociated into MMs under the conditions used for the SDS gel electrophoresis (Fig. 3A, B). Western blots against HtrA and UreB served as loading controls (Fig. 3C). As another control, we confirmed that the various HtrA mutations did not affect the bacterial growth characteristics over time (Fig. 3D) and survival of the bacteria at different temperatures (37 °C, 39 °C or 41 °C) (Fig. 3E, Fig. S5A) or under varying pH stress conditions (pH 7.1 or 5.2) (Fig. 3F, Fig. S5B). In all experiments, the *htrA* knockout strain was used as a negative control.

**Fig. 4:**
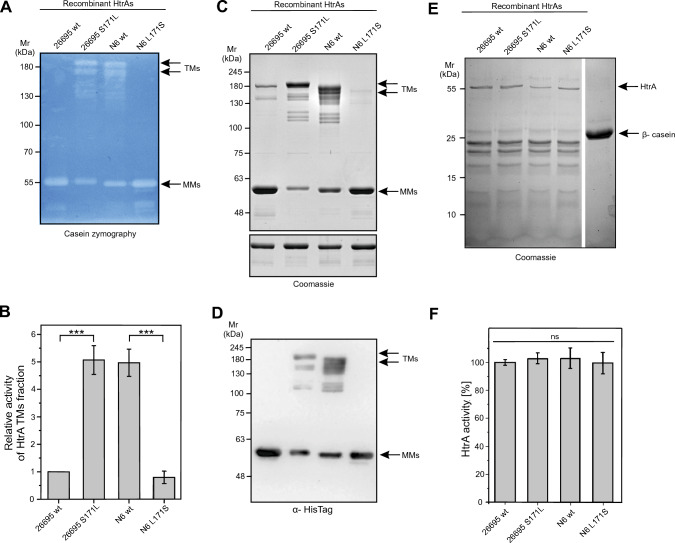
Mutation analysis of recombinant *H. pylori* HtrA variants reveals changes in TM stability. **A** Casein zymography showing the proteolytic activity and TM stability of 100 ng recombinant wild-type *H. pylori* HtrAs and corresponding point mutations at position 171. **B** The relative activity of HtrA TM fraction. The activity of HtrA^26695^ ^wt^ was set to 1*.* The error bars represent the standard error of the mean (SEM) from four independent experiments*.* **C** The upper panel shows 2 µg of non-denatured HtrA recombinant proteins that were separated using standard SDS-PAGE (without casein) to observe TM stability. Bottom panel showed 1 µg of heat-denatured HtrA recombinant proteins that were separated to confirm similar amounts of protein per lane. **D** 300 ng of non-denatured HtrA recombinant proteins analyzed by Western blotting. **E** Effect of HtrA point mutations on the cleavage of β-casein. Examples of representative experiments are shown. **F** Effect of point mutations on the proteolytic activity of HtrA at 37 °C and pH 6.2. Proteolytic activity of HtrA was tested using β-casein as a substrate. The proteolytic activity of HtrA^26695^ ^wt^ was set to 100%. The molar ratio of HtrA/β-casein was 1:100

**Fig. 5:**
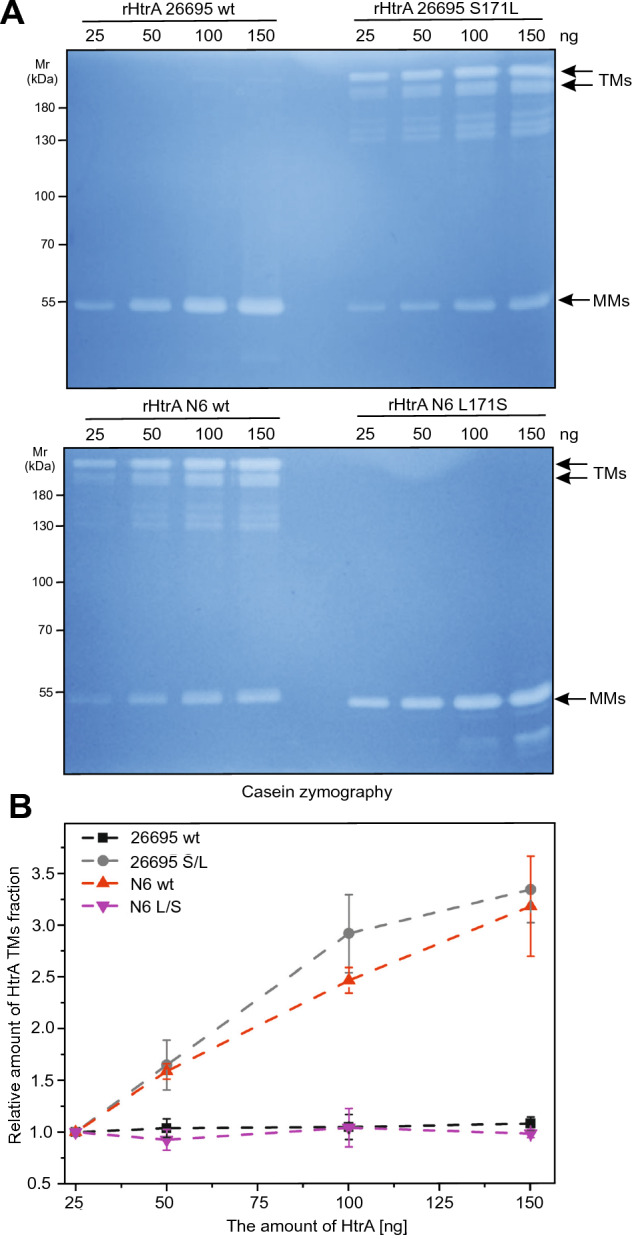
The ability to maintain *H. pylori* HtrA TMs is not affected by varying protein concentrations. **A** Casein zymography of increasing amounts of recombinant HtrA. **B** The relative amount of HtrA activity in the TM fraction was analysed densitometrically. The values of 25 ng of each of the HtrAs were set to 1*.* The error bars represent the standard error of the mean (SEM) from four independent measurements

**Fig. 6:**
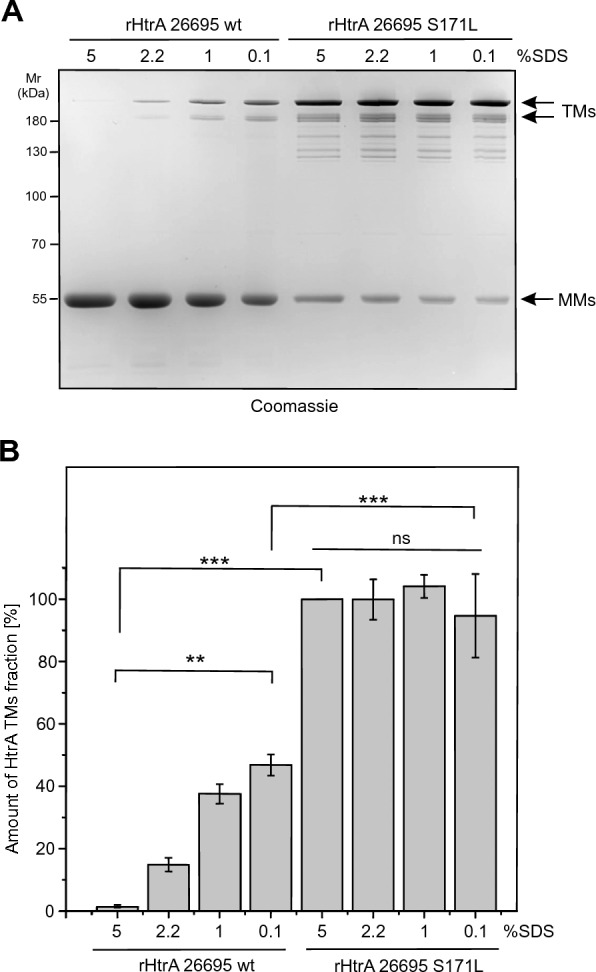
The ability to maintain *H. pylori* HtrA TMs is affected by SDS concentration. **A** The gel shows 2 µg of non-denatured HtrA recombinant proteins that were separated using standard SDS-PAGE (without casein). The 2.2% concentration of SDS is used in standard SDS buffer without β-mercaptoethanol (casein buffer). **B** The relative amount of HtrA TM fraction was analysed densitometrically. The amount of rHtrA 26695 S171L incubated with 5% SDS was set to 100%*.* The error bars represent the standard error of the mean (SEM) from three independent experiments

Furthermore, we purified the recombinant His-tagged HtrAs of 26695 wt, 26695^S171L^, N6 wt and N6^L171S^. In agreement with the results shown in Fig. 3A, B, we observed the same effect of the HtrA 171S/L mutation on TM stability of the corresponding purified recombinant proteins in zymograms, Coomassie-stained gels and Western blots (Fig. 4A–D). Under the given conditions, we found a similarly high proteolytic activity of the HtrA variants against the artificial substrate β-casein (Fig. 4E, F). As a further control, TM stability was not affected by differences in the HtrA concentration (Fig. 5). We also analysed the impact of the anionic surfactant sodium dodecyl sulfate (SDS) on TM stability of HtrA using SDS-PAGE. SDS interacts with proteins to form a negatively charged SDS-protein complex. Importantly, the hydrophobic interactions, ionic bonds, and hydrogen bonds could be interrupted by this surfactant component [45]. We used controlled concentrations of SDS (5%; 2.2%; 1% and 0.1%) in SDS-casein buffer, in which the tested proteins were resuspended, before loading the samples into the wells. The protein samples were not boiled. We observed that with increasing SDS concentration, the stability of recombinant HtrA of 26695 TMs decreased, in contrast to recombinant HtrA of 26695 S171L, which showed a constant presence of TMs under the tested conditions (Fig. 6A, B). Even at the highest concentration of SDS, the S171L HtrA effectively maintained stable TMs. However, the presence of a serine at the tested position caused an extreme reduction in the amount of detected TMs (Fig. 6A, B). Taken together, while HtrAs containing 171L formed stable TMs, HtrAs with the 171S allele did not and were predominantly present in the MM fraction. Thus, the *H. pylori* phenomenon concerning HtrA TM stabilization is genetically determined by the natural L/S171 polymorphism in clinical strains.

### Variability of the studied amino acid position in HtrAs from other bacteria

The L/S171 polymorphism results in the exchange of a hydrophobic by a polar amino acid. To assess the putative consequences of this type of exchange, we investigated the biophysical properties of the amino acids present at the respective sequence position in other bacterial HtrAs. Sequence alignment of *H. pylori* HtrA with that of representative other bacterial pathogens showed that the studied region of the proteolytic domain is quite well conserved during evolution. However, position 171 itself exhibits a rather large sequence variability (Fig. S6, red asterisk). The overwhelming majority of HtrAs revealed a hydrophilic amino acid at the studied position, including arginine (*Salmonella, Klebsiella, Shigella, Yersinia, Proteus, Vibrio, Haemophilus, Bacillus* and *Escherichia* species), lysine (*Neisseria, Listeria* and *Clostridium* species, as well as *Staphylococcus epidermidis*), glutamic acid (*Legionella* species), serine (*Streptococcus oralis*), or threonine (*Streptococcus mitis*)*.* One exception found was a hydrophobic valine in *Staphylococcus aureus*. The importance of the biophysical properties at position 171 for the maintenance of HtrA TMs has not yet been investigated. The published casein zymograms for HtrA (or its homolog DegQ) from numerous bacterial species are available in the literature: *Salmonella* typhimurium [44], *Yersinia enterolytica* [44]*, Escherichia coli* [44]*, Proteus mirabilis* [44]*, Neisseria gonorrhoeae* [17], *Shigella flexneri* [17] and *Listeria monocytogenes* [46]. For all these HtrAs, which have a hydrophilic amino acid at the tested position, TM instability can be observed in the corresponding zymograms.

Interestingly, an evolutionarily related bacterium of *H. pylori*, the intestinal pathogen *Campylobacter jejuni*, exhibits a methionine at position 171 of its HtrA (Fig. S7A, red asterisk). Using casein zymography, we tested various available *C. jejuni* wt strains from our strain collection including NCTC11168, 81-176, 81116, 84-25, 43431 and RM1221, with the NCTC11168Δ*htrA* knockout mutant serving as the negative control. Similar to the L-type *H. pylori* strain N6, all tested *C. jejuni* wt isolates formed stable HtrA TMs (Fig. S7B). Methionine, like leucine, belongs to the hydrophobic amino acids. Thus, it appears that hydrophobic amino acids at position 171 of bacterial HtrAs, such as leucine or methionine in case of *H. pylori* and *C. jejuni*, favor HtrA TM stability. Future studies should investigate in detail the molecular basis and possible clinical consequences of this remarkable polymorphism in regulating the proteolytic activity of bacterial HtrAs.

## Discussion

*Helicobacter pylori* represents the first described bacterial carcinogen implicated in gastric cancer, but also accounting for the development of other gastric diseases such as chronic gastritis and peptic ulceration [1–3, 47]. In addition to the major virulence factors CagA and VacA, serine protease HtrA emerges as a remarkable new pathogenic protein of *H. pylori*. During infection, this secreted protease cleaves the tumour suppressor E-cadherin in the adherens cell-to-cell junctions as well as claudin-8 and occludin in the tight junctions, which causes severe damage in the infected gastric epithelium [9, 11–13, 17–19]. In the present study, we discovered 12 natural mutations in the *htrA* gene that resulted in an amino acid exchange in the HtrA protein (Fig. 2). Of particular interest was one mutation in the HtrA protease domain at position 171 that correlated with TM maintenance in clinical isolates (Figs. 1, 2). Importantly, we showed that the 171L variant (but not 171S) formed stable HtrA TMs in the bacterial cells in vivo (Figs. 1 and 3) as well as in the form of purified recombinant HtrA in vitro (Fig. 4).

It is well-established for many years that HtrAs from bacteria assemble as active homo-TMs, representing the principal structural units [48]. In various bacteria these TMs can even generate higher-order oligomers, which differ in size and coherence mechanisms depending on the bacterial species [49–51]. The primary model system in bacterial HtrA research is DegP from *Escherichia coli* [49]*.* The inactive configuration of DegP is the hexamer, which upon activation, can dissociate to single TMs. As long as cleavable substrate proteins are available for the DegP enzyme, its TMs are proteolytically active and can even successively form higher active multimers such as 12-mers and 24-mers. When the proteolysis of target proteins is finished, DegP converts again to inactive hexamers [52]. However, our previous data showed that *H. pylori* HtrAs can form caseinolytically active TMs, which under some conditions are stable in vitro, for example during size exclusion chromatography [12]. In addition, analytical ultracentrifugation data showed that higher oligomers of strain 26695 (with 171S-type HtrA) are unstable, in contrast to strain N6 (with 171L-type HtrA) that exhibits highly stable TMs and even higher oligomeric forms [12]. Our further data demonstrated that the N-terminus of HtrA is crucial for the stable trimerization of HtrA [15]. In particular, we detected two N-terminal cleavage sites in HtrA (between H 46/D47 and K50/D51), and inactivation of these cleavage sites by mutagenesis revealed the loss of certain interactions among the individual HtrA subunits, resulting in destabilization of the TM associated with loss of enzymatic activity [15]. Structural analyses and casein zymography gels indicated that *H. pylori* HtrA lacking the entire N-terminal part is unable to form TMs and that the resulting MMs are proteolytically inactive [14, 15]. In the present paper, we used zymography and monitored the enzymatic activity of full-length HtrA by casein cleavage, visualized as white bands (caused by complete digestion of casein) in both the TM and MM fractions. This observation suggests that *H. pylori* full-length HtrA MMs, in contrast to the N-terminally truncated HtrA [14, 15], might still retain an active conformation and possess residual caseinolytic activity. Alternatively, a dynamic equilibrium between MMs and TMs in the MM fraction may emerge after the removal of SDS from the gels and HtrA-SDS complex, which would explain the band at ~ 55 kDa by the proteolytically active TMs in this fraction.

Taken together, we identified the amino acid at position 171 as crucial for maintaining HtrA TMs. However, using casein as an artificial substrate, we detected a strong caseinolytic activity, but no significant differences among the activities of HtrA in the MM and TM fractions. The reason for this finding is still unclear. Experiments are currently underway in our lab investigating the importance of the L/S171 mutation in HtrA-mediated cleavage of cell junction proteins occludin, claudin-8 and E-cadherin during infection. Previous studies investigated potential ligand and inhibitor binding sites on the surface of *H. pylori* HtrA [53, 54]. A mutation analysis showed that insertion of single S164A, S166A, N208A and K328A amino acid changes near the active centre of the protease domain in HtrA leads to an inability to cleave E-cadherin. These observations support the idea that these residues may play relevant roles in the functional regulation of HtrA [54]. The natural L/S171 mutation found here is in the close vicinity of the S164 and S166 residues (Fig. 2). In addition, it was reported that seven *H. pylori*-positive patients (with HtrA-L171 allele) obtained from patients non-ulcer dyspepsia, revealed elevated serum gastrin values compared to eleven patients carrying HtrA-S171 isolates, which could be involved in gastric disease outcome [55]. However, all the latter strains were not isogenic and revealed various other HtrA mutations, for example including polymorphisms at positions 6F/L, 25N/S, 68S/N, 303 V/I, 312A/V and 382 T/A with unknown impact. Interestingly, among the HtrAs of various important bacterial pathogens, hydrophilic amino acid residues (serine, threonine, arginine, lysine, and glutamic acid) are the predominant ones at position 171, whereas hydrophobic amino acids (leucine, methionine) are found in *H. plyori* and *C. jejuni* (Figs. S6 and S7). Thus, it clearly requires more experiments to investigate how relevant L/S171 and other mutations can be for the overall regulation of *H. pylori* HtrA stability and proteolytic activity during infection. In conclusion, our current study revealed a crucial role of amino acid position 171 in *H. pylori* HtrA for TM stabilization of the protease enzyme. Thus, this mutation could be a potential genetic biomarker for *H. pylori*-triggered gastric diseases, which deserves further detailed investigations.

## Supplementary Information

Below is the link to the electronic supplementary material.

Supplementary file1 (DOCX 6493 KB)

## Data Availability

All data are available.
