# Supplementary material for: Trimer stability of Helicobacter pylori HtrA is regulated by a natural mutation in the protease domain
Source: Med Microbiol Immunol. 2023 May 14;212(3):241–52. doi: 10.1007/s00430-023-00766-9 (PMC10293373; doi:10.1007/s00430-023-00766-9)
Supplement: Supplementary file 1 — Supplementary file1 (DOCX 6493 KB) [file 430_2023_766_MOESM1_ESM.docx]

**Supplementary Data**

Medical Microbiology and Immunology

**Trimer stability of *Helicobacter pylori* HtrA is regulated by a natural mutation in the protease domain**

Urszula Zarzecka^1,2^, Nicole Tegtmeyer^1^, Heinrich Sticht^3^, and Steffen Backert^1,§^

^1^Division of Microbiology, Department of Biology, Friedrich-Alexander-Universität Erlangen-Nürnberg, Erlangen, Germany

^2^Department of General and Medical Biochemistry, Faculty of Biology, University of Gdańsk, Gdańsk, Poland

^3^Division of Bioinformatics, Institute of Biochemistry, Friedrich-Alexander-Universität Erlangen-Nürnberg; Erlangen, Germany

^§^Corresponding author: [Steffen.Backert@fau.de](mailto:Steffen.Backert@fau.de), +49-9131-8528081


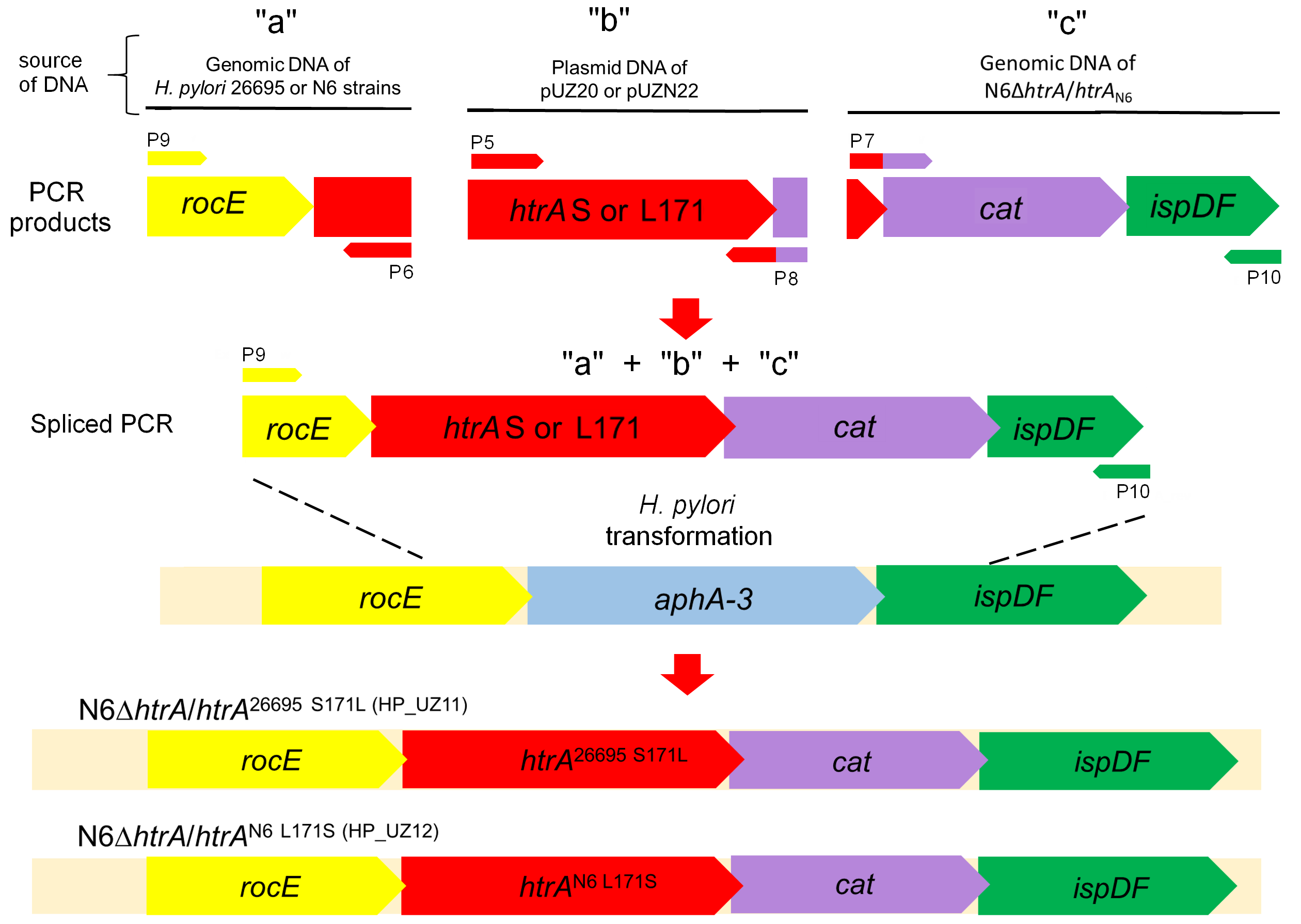


**Supplementary Figure S1.** Schematic presentation of gene splicing by overlap extension PCR (SOE-PCR) strategy used to construct *H. pylori* mutants carrying various substitutions in the *htrA* gene such as S171 and L171S (N6 ∆*htrA/htrA*^26695 S171L^ and N6 ∆*htrA/htrA*^N6 L171S^). Utilized primer sequences are provided in Table S2.


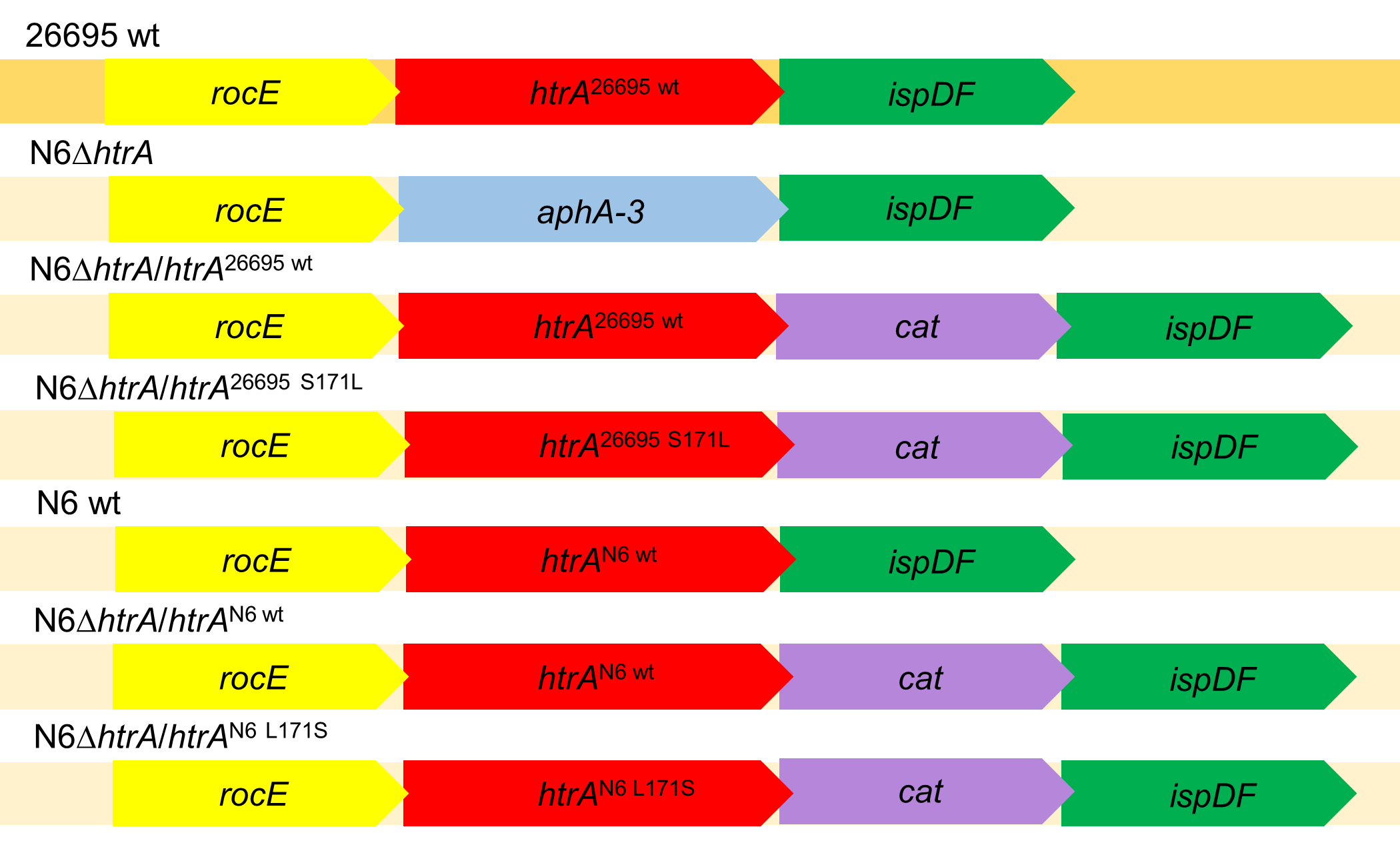


**Supplementary Figure S2.** Schematic presentation of the *htrA* locus and the flanking chromosomal regions in the *H. pylori* mutant strains produced in this study: wt (N6 and 26695), Δ*htrA*, two complemented strains (Δ*htrA*/*htrA*^N6^ ^wt^ and Δ*htrA*/*htrA*^26695 wt^) and two strains with swab mutations at 171 position, which were selected as a crucial for trimer stability (Δ*htrA*/*htrA*^N6^ ^L171S^ and Δ*htrA*/*htrA*^26695 S171L^).


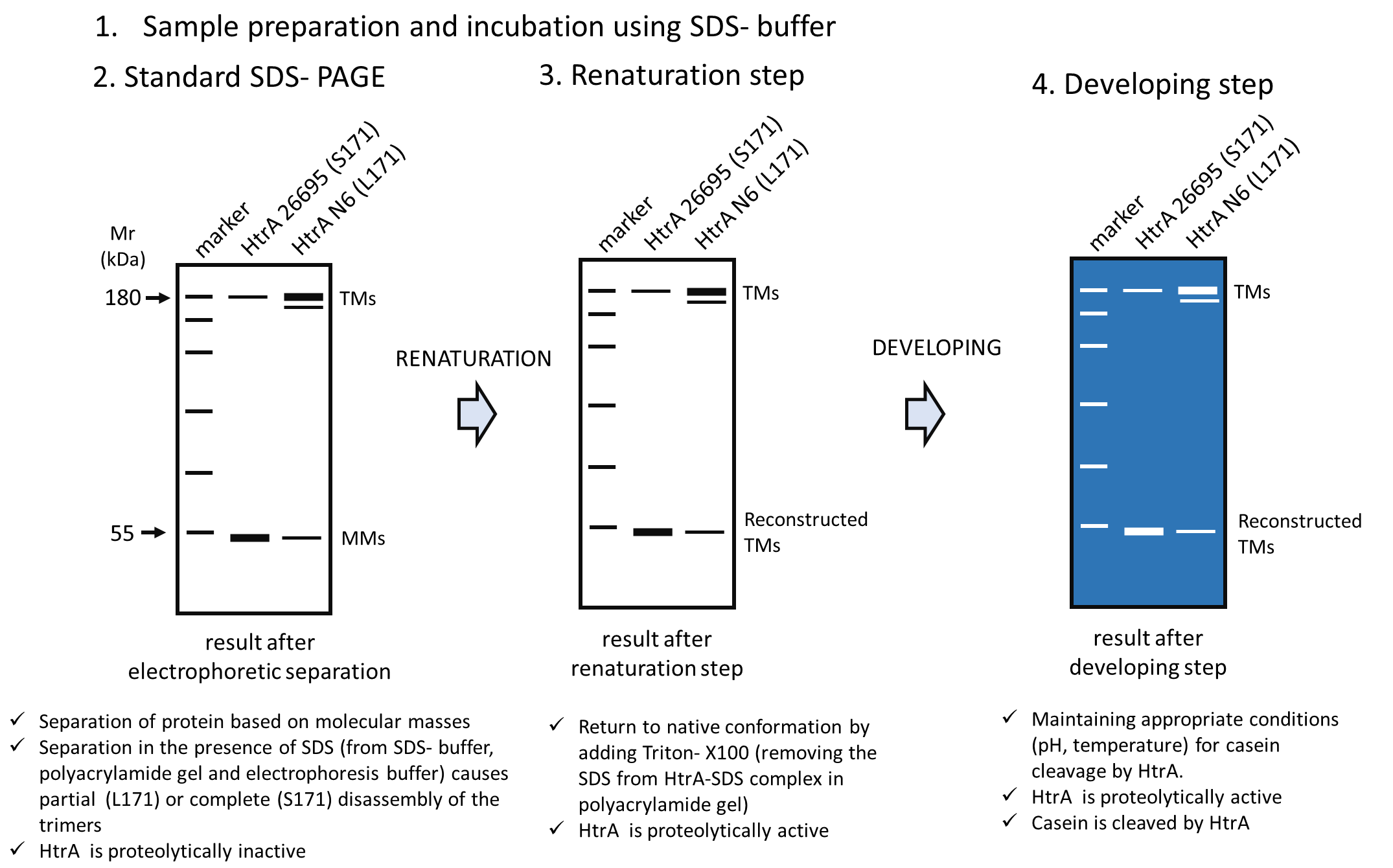


**Supplementary Figure S3.** **Schematic presentation explaining the methodology used in this report to study HtrA trimer stability by casein zymography.** The various stages of the method and the most significant effects of using certain reagents are shown. First, the protein samples of interest are suspended in SDS-containing buffer without β-mercaptoethanol and, importantly, the boiling stage is omitted (Step 1). Subsequently, the proteins are separated under denaturing conditions (maintained by SDS) according to size and molecular mass by SDS-PAGE. Electrophoretic separation allows the detection of two fractions of HtrA: trimers (~180 kDa, TMs) and monomers (~55 kDa, MMs) as indicated, but under these conditions all separated proteins are inactive (Step 2). A renaturation step follows by adding 2.5% Triton-X100 enabled the removal of SDS from the protein-SDS complex in the gel, which initiated the process of renaturation, and recovery of the native HtrA structure and its activity (Clarke 1981). We propose that after removal of SDS, HtrA in the gel formed and maintained a trimer structure both at the monomer and trimer fractions, respectively (Step 3). In the developing phase, we change the conditions (temperature and pH) to those optimal for casein cleavage as described in Materials & Methods. Finally, Coomassie Blue staining reveals white bands in the gels, which are indicative of the cleavage of casein by HtrA (Step 4).


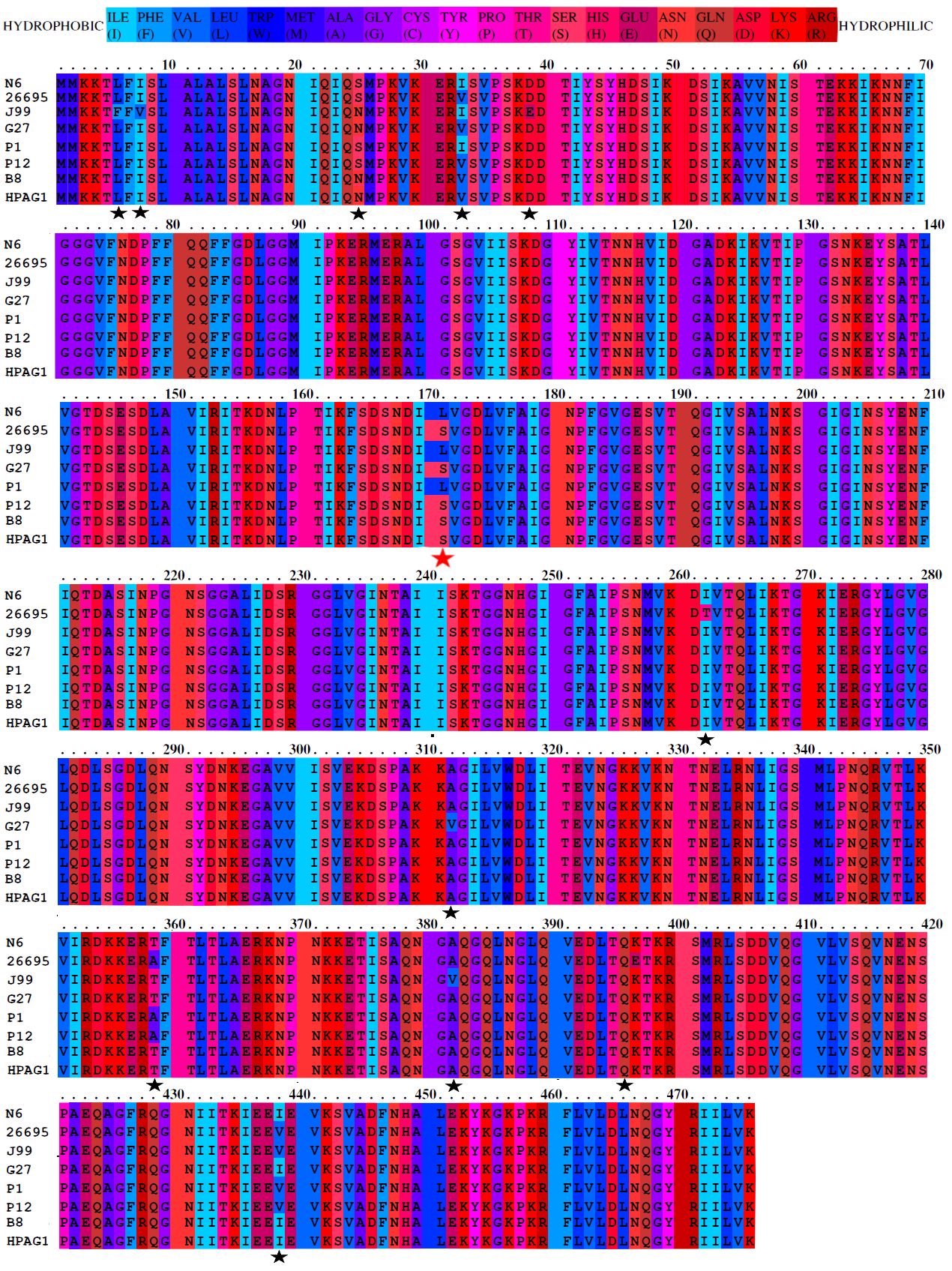


**Supplementary Figure S4**. **Amino acid hydrophobicity properties of serine protease HtrA from various clinical *H. pylori* strains.** The sequences were aligned with the Praline tool of the IBIVU server (<https://www.ibi.vu.nl/programs/pralinewww/>; [41]). The hydrophobicity scale used is from [42]. Black asterisks indicate the position of variable amino acid residues. The red asterisk indicates amino acid position 171 that is important for trimer stability. Significant changes in hydrophobicity were found at positions 171, 263 and 359. The positions 171 and 263 are located in the protease domain. The hydrophobicity of the amino acid residues plays an important role in the determination of protein structure, interactions, and function.


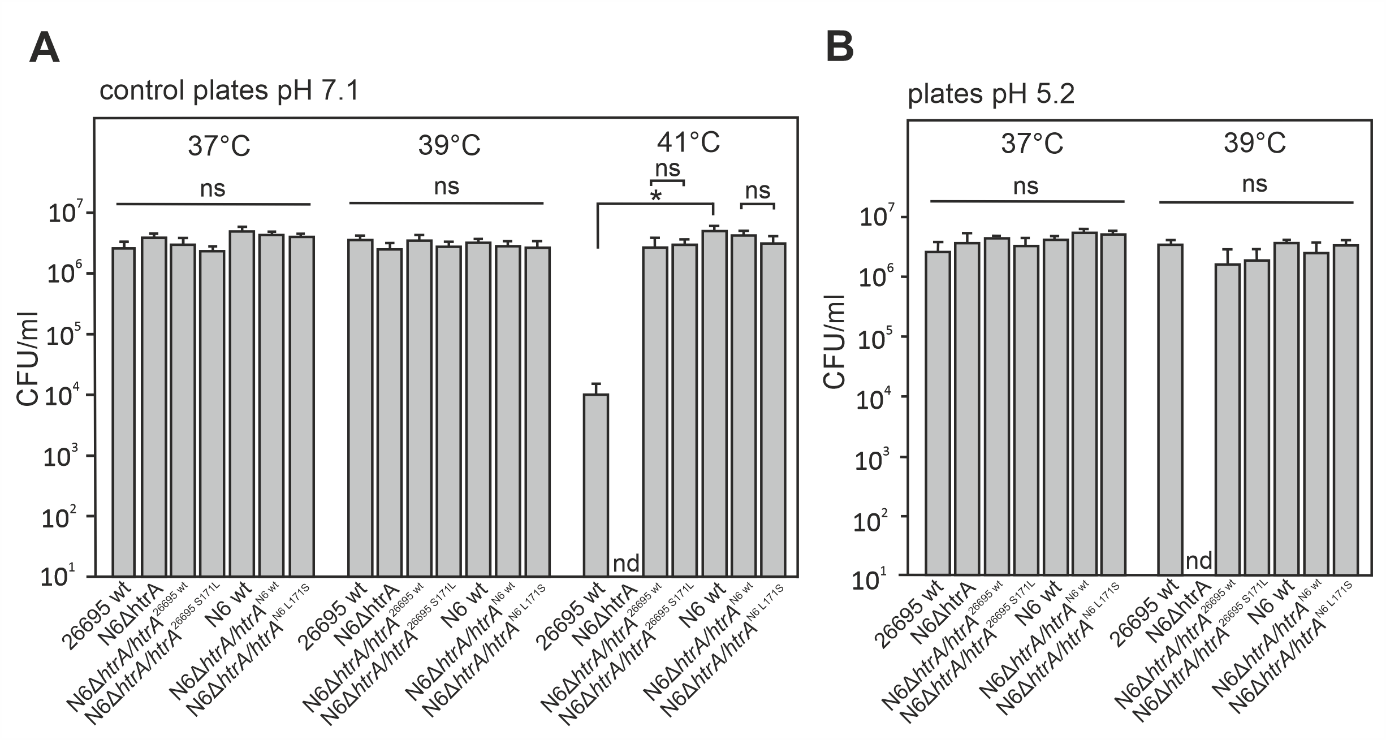


**Supplementary Figure S5. Quantification of *H. pylori* survival rates caused by heat and pH stress.** The indicated *H. pylori* bacteria were grown on GC agar plates under various stress conditions caused by temperature (37°C, 39°C and 41°C) and pH (5.2 vs. 7.1, respectively). Panels **(A)** and **(B)** correspond to Figure 4 panels D and E, respectively. The standard error of mean (SEM) was calculated using at least three repetitions. Data were evaluated using the Bonferroni test. Statistical significance was defined by *p ≤ 0.05* (*). Nd - no single colonies detected; ns- no significant differences.


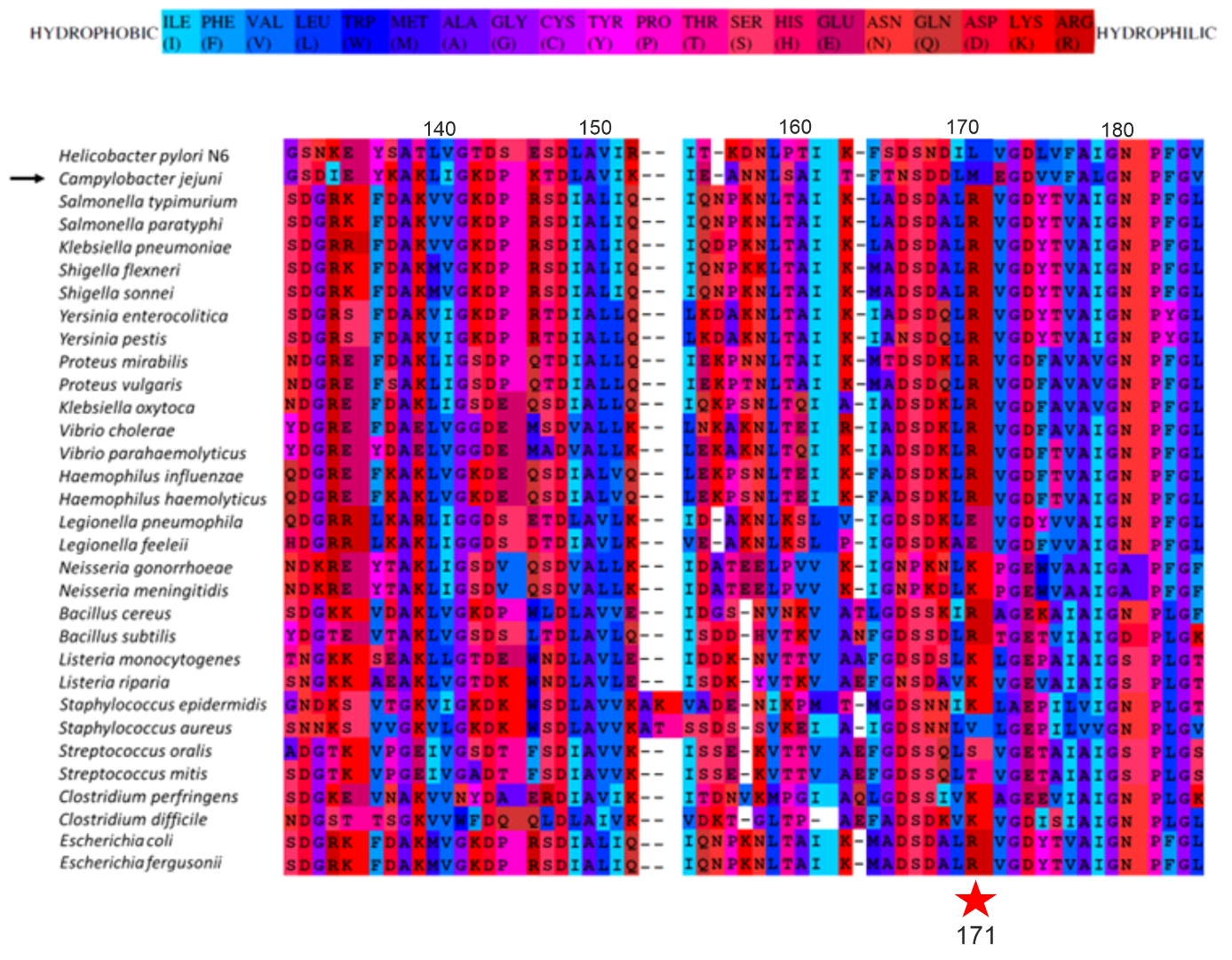


**Supplementary Figure S6**. **Amino acid hydrophobicity properties of HtrAs from various bacterial species.** The sequences were aligned with the Praline tool of the IBIVU server (<https://www.ibi.vu.nl/programs/pralinewww/>; [41]). The hydrophobicity scale used is from [42]. The red asterisk indicates amino acid position 171 for HtrA of *H. pylori* from N6 strain. The numbering of amino acid residues is given for HtrA of *H. pylori* N6 strain.


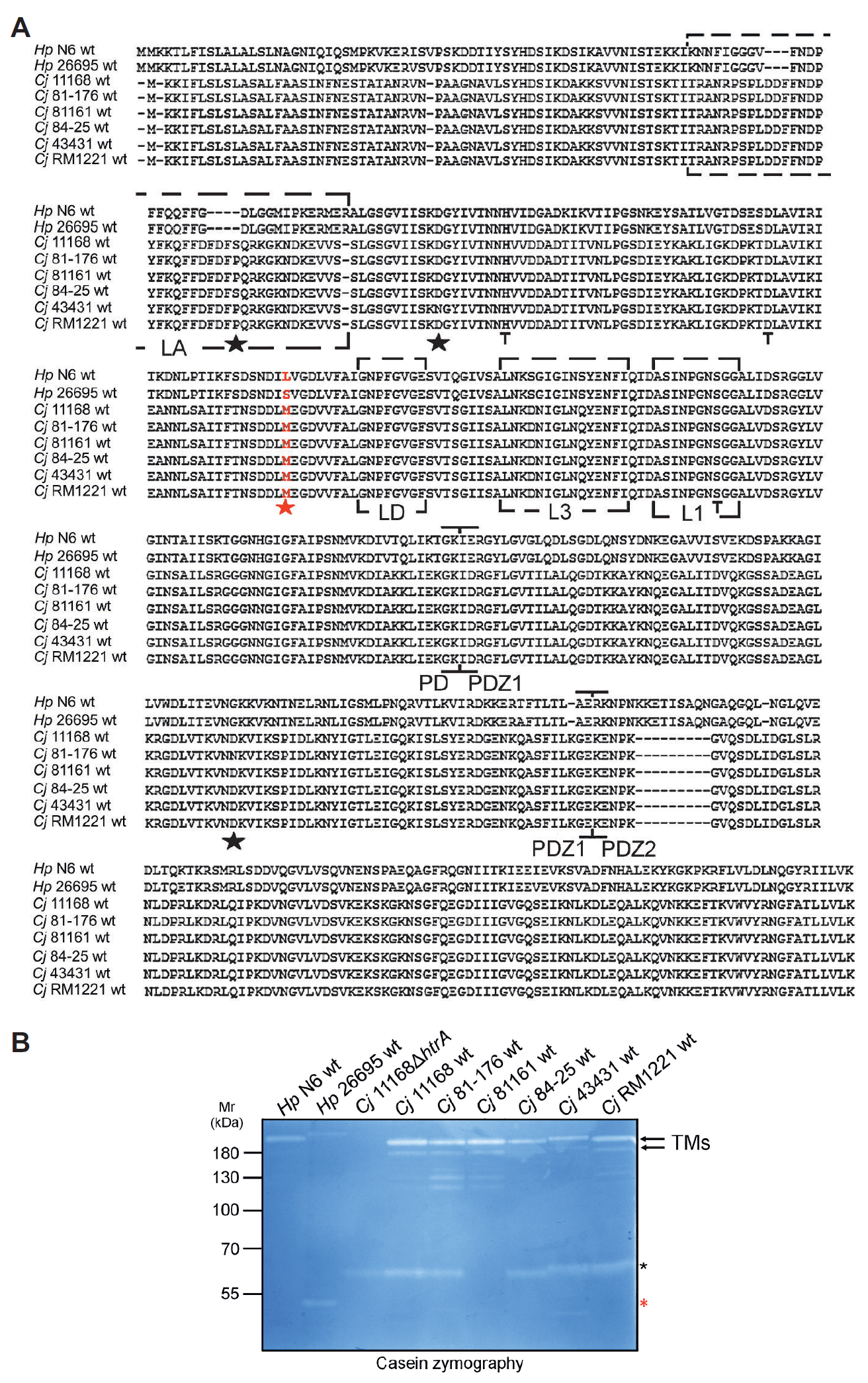


**Supplementary Figure S7. Sequence analysis and proteolytic activity of HtrA from various *C. jejuni* strains. (A)** Comparison of the amino acid sequences of HtrAs from various indicated *H. pylori* and *C. jejuni* strains. The important regulatory loops (LA, LD, L1, L2, L3) as well as domain organization are marked with dashed lines. PD- stands for the protease domain, T- catalytic triad. Black asterisks indicate the differences between the HtrAs of *C. jejuni* strains. The red asterisk represents the position of 171 for *H. pylori* HtrA. **(B) Proteolytic activity of HtrAs from the indicated bacterial lysates w**as tested using casein zymography. The trimer fraction (TMs) is indicated with arrows. The C. jejuni 11168∆htrA was used as a control. Black asterisk labels the position of other proteolytically active protein bands. The red asterisk labels the position of HtrA monomer. The black asterisk marks a caseinolytically active peptidase unique to *C. jejuni*, which was recently identified as PepP (Heimesaat et al. 2020).

**Table S1.** Bacterial strains and plasmids used in the current study.

| **Strain/plasmid** | **Genotype** | **Reference/source** |
| --- | --- | --- |
| *E. coli* BL21DE3 | F^-^ *ompT hsdS_B_(r_B_ ^–^m_B_^-^) gal dcm* | Novagen |
| *H. pylori* Ka36 | Wild type strain | [22] |
| *H. pylori* Ka61 | Wild type strain | [22] |
| *H. pylori* Ka77 | Wild type strain | [22] |
| *H. pylori* Ka86 | Wild type strain | [22] |
| *H. pylori* Ka88 | Wild type strain | [22] |
| *H. pylori* Ka89 | Wild type strain | [22] |
| *H. pylori* Ka92 | Wild type strain | [22] |
| *H. pylori* Ka95 | Wild type strain | [22] |
| *H. pylori* Ka98 | Wild type strain | [22] |
| *H. pylori* Ka112 | Wild type strain | [22] |
| *H. pylori* Ka125 | Wild type strain | [22] |
| *H. pylori* Ka148-1 | Wild type strain | [22] |
| *H. pylori* Ka148-2 | Wild type strain | [22] |
| *H. pylori* Ka161 | Wild type strain | [22] |
| *H. pylori* Ka169 | Wild type strain | [22] |
| *H. pylori* Ka171 | Wild type strain | [22] |
| *H. pylori* Ka192 | Wild type strain | [22] |
| *H. pylori* Ka204 | Wild type strain | [22] |
| *H. pylori* Ka223 | Wild type strain | [22] |
| *H. pylori* Ka226 | Wild type strain | [22] |
| *H. pylori* UH1 | Wild type strain | [22] |
| *H. pylori* UH4 | Wild type strain | [22] |
| *H. pylori* UH44 | Wild type strain | [22] |
| *H. pylori* Ca38 | Wild type strain | [22] |
| *H. pylori* Ca70 | Wild type strain | [22] |
| *H. pylori* Ca71 | Wild type strain | [22] |
| *H. pylori* Ca81 | Wild type strain | [22] |
| *H. pylori* Ca117 | Wild type strain | [22] |
| *H. pylori* Ca130 | Wild type strain | [22] |
| *H. pylori* Ca169 | Wild type strain | [22] |
| *H. pylori* Ca178 | Wild type strain | [22] |
| *H. pylori* Ca205 | Wild type strain | [22] |
| *H. pylori* 26695 | Wild type strain | [23] |
| *H. pylori* J99 | Wild type strain | [24] |
| *H. pylori* G27 | Wild type strain | [25] |
| *H. pylori* P1 | Wild type strain | [26] |
| *H. pylori* P12 | Wild type strain | [27] |
| *H. pylori* B8 | Wild type strain | [28] |
| *H. pylori* HPAG1 | Wild type strain | [29] |
| *H. pylori* N6 | Wild type strain | [30] |
| N6 ∆*htrA* | *H. pylori* N6 *secA*R837K ∆*htrA, Kan^R^* | [13] |
| N6 ∆*htrA/htrA*^N6 wt^ | *H. pylori* N6 *secA*R837K *∆htrA*/*htrA*N6 wt, Cm^R^ | [13] |
| HP_UZ1/ N6 ∆*htrA/htrA*^26695 wt^ | *H. pylori* N6 *secA*R837K *∆htrA*/*htrA2*6695 wt, Cm^R^ | [31] |
| HP_UZ11/ N6 ∆*htrA/htrA*^26695^ ^S171L^ | *H. pylori* N6 *secA*R837K *∆htrA*/*htrA2*6695 S171L, Cm^R^ | This work |
| HP_UZ12/ N6 ∆*htrA/htrA*^N6^ ^L171S^ | *H. pylori* N6 *secA*R837K *∆htrA*/*htrA*N6 L171S, Cm^R^ | This work |
| C. jejuni NCTC11168 | Wild type strain | [32] |
| C. jejuni NCTC11168∆*htrA* | C. jejuni NCTC11168 ΔhtrA, Cm^R^ | [32] |
| *C. jejuni* 81-176 | Wild type strain | [33] |
| *C. jejuni* RM1221 | Wild type strain | [33] |
| *C. jejuni* 84-25 | Wild type strain | [34] |
| *C. jejuni* 81116 | Wild type strain | [35] |
| C. jejuni ATCC 43431 | Wild type strain | [36] |
| pHJS5 | pET26b, wt *htrA* from the *H. pylori* 26695 strain with C- terminal His_6_-tag, Kan^R^ | [12] |
| pUZ20 | pET26b, *htrA* S171L from the *H. pylori* 26695 strain with C- terminal His_6_-tag, Kan^R^ | This work |
| pUZN10 | pET26b, wt *htrA* from the *H. pylori* N6 strain with C- terminal His_6_-tag, Kan^R^ | [13] |
| pUZN22 | pET26b, *htrA* L171S from the *H. pylori* N6 strain with C- terminal His_6_-tag, Kan^R^ | This work |

**Table S2.** PCR primers used in the present studies.

| **Number** | **Orientations** | **Sequence (5`> 3`)** |
| --- | --- | --- |
| P1 | Forward | GATTCTAATGATATTTTAGTGGGCGATTTGGT |
| P2 | Reverse | CAAATCGCCCACTAAAATATCATTAGAATCAGAGA |
| P3 | Forward | TGATTCTAATGATATTTCAGTGGGCGATTTGGT |
| P4 | Reverse | CAAATCGCCCACTGAAATATCATTAGAATC |
| P5 | Forward | CTTTAGCGTTAAGCTTGAATGCGGGCAATATCCAAATCCAG |
| P6 | Reverse | CTGGATTTGGATATTGCCCGCATTCAAGCTTAACGCTAAAG |
| P7 | Forward | GGATCATTTTGGTGAAATGACTAACTAGGAAGCTAAAATGGAGAAAA |
| P8 | Reverse | TTTTCTCCATTTTAGCTTCCTAGTTAGTCATTTCACCAAAATGATCC |
| P9 | Forward | TAATGTGATCAGTTTCACGG |
| P10 | Reverse | GGCGTTTGATGTAAATATAA |

**Table S3.** Primers and template DNAs used in the strategy for obtaining the required PCR products and plasmid constructs.

| **Step 1** | | | | | | | |
| --- | --- | --- | --- | --- | --- | --- | --- |
| PCR product “a” | | PCR product “b” | | | PCR product “c” | | |
| **Primer pair 1** | **Template** | **Primer pair 2** | | **Template** | **Primer pair 3** | | **Template** |
| P6; P9 | Genomic DNA of Hp 26695 | P5; P8 | | Plasmid DNA pUZ20 | P7; P10 | | Genomic DNA of N6 ∆*htrA/htrA*^N6 wt^ |
| P6; P9 | Genomic DNA of Hp N6 | P5; P8 | | Plasmid DNA of pUZN22 | P7; P10 | | Genomic DNA of N6 ∆*htrA/htrA*^N6 wt^ |
| **Step 2** | | | **The resulting construct** | | |  |  |
| S-PCR fragment | | |  |  |  |  |  |
| **Primer pair** | **Feature** | |  |  |  |  |  |
| P9; P10 | htrA^26695^ ^S171S^ | | N6 ∆*htrA/htrA*^26695 S171L^ | | |  |  |
| P9; P10 | htrA^N6^ ^L171S^ | | N6 ∆*htrA/htrA*^N6^ ^L171S^ | | |  |  |

**Supplementary References**

1. Heimesaat MM, Schmidt AM, Mousavi S, Escher U, Tegtmeyer N, Wessler S, Gadermaier G, Briza P, Hofreuter D, Bereswill S, Backert S. (2020) Peptidase PepP is a novel virulence factor of *Campylobacter jejuni* contributing to murine campylobacteriosis. Gut Microbes. 12(1):1770017. doi: 10.1080/19490976.2020.1770017.
2. Clarke S (1981) Direct renaturation of the dodecyl sulfate complexes of proteins with triton X-100. Biochimica et Biophysica Acta (BBA) - Protein Structure 670:195–202. https://doi.org/10.1016/0005-2795(81)90009-X.
